# Internet of things enabled deep learning methods using unmanned aerial vehicles enabled integrated farm management

**DOI:** 10.1016/j.heliyon.2023.e18659

**Published:** 2023-07-26

**Authors:** Shailendra Mishra

**Affiliations:** Department of Computer Engineering, College of Computer and Information Sciences, Majmaah University, Al Majmaah, 11952, Saudi Arabia

**Keywords:** Smart livestock farming, Internet of things, Deep learning, Unmanned aerial vehicles, Integrated farm management

## Abstract

Smart livestock farming strives to make farming more lucrative, efficient, and ecologically beneficial by using digital technologies. Precision livestock fencing, in which each animal is followed and studied independently, is the most promising kind of smart livestock farming. The Internet of Things (IoT) allows farmers to save money and effort by keeping tabs on crops, mapping out their land, and giving them data to develop sensible management strategies for their farms. Surveillance, disaster management, firefighting, border patrol, and courier services employ Unmanned Aerial Vehicles (UAVs) that are originally created for the military. The segment focuses on UAVs in livestock and agricultural production. This is achieved via employing robots, drones, remote sensors, and computer imagery in unison with ever-improving in-Depth Learning for farming. Deep learning (DL) algorithms find many uses in the agricultural sector, from identifying plant diseases to estimating yields to detecting weeds to forecasting the weather and determining how much water is in the soil. The challenging characteristics of smart livestock farming are climate change, biodiversity loss, and continuous monitoring. Hence, in this research, the Unmanned Aerial Vehicles enabled Integrated Farm Management (UAV-IFM) has been designed to improve smart livestock farming. Safe and reliable tracking of livestock from farm to fork is made possible by this sensor, which has far-reaching implications for detecting and containing disease outbreaks and preventing the resulting financial losses and food-related health pandemics. UAV-IFM aims to improve the assessment process so that smart livestock farming may be more widely adopted and offers growth-supportive help to farmers. Conclusions gathered from this study's examination of the UAV-IFM reveal that these instruments correctly forecast and verify smart livestock farming management within the framework of the assessment procedure. The experimental analysis of UAV-IFM outperforms smart livestock farming in terms of efficiency ratio, performance, accuracy, and prediction.

## 1 Overview of smart livestock farming usage and its impact

Farmers tend on several animal species in agricultural fields. Checking on cattle manu-ally might be a hassle since they tend to wander about [1]. The concept of “smart farming” owes its origins to the introduction of new agricultural and livestock production technology during the Industrial Revolution, which made it possible to increase output while decreasing waste and pollution [2]. Data from the farming operation can be recorded, critical informa-tion extracted, and in some cases, the product. The livestock is the product to which the authors refer. Farmers who employ smart livestock farming methods can track a variety of operational metrics, including but not limited to animal health and productivity, as well as extract actionable insights from the data. Farmers can improve the health, productivity, and profitability of their livestock by using data collected from their operations to inform better decisions about the care and management of their livestock. Farmers can use this information to take preventative measures against health problems and gain insights into the effect environmental factors have on the livestock's well-being.

Finite resources, such as land, must be managed, as must the need to lessen livestock's impact on greenhouse gases and the management of highly contextual and repetitive day-to-day tasks involved in livestock management [3,4].

The Internet of Things (IoT) is a new technological paradigm enhancing everyday items with computational intelligence. Healthcare, utility infrastructure, and farming are just a few examples of fields where IoT might be beneficial. Utilizing the Internet of Things allows us to optimize previously complicated processes that required a lot of resources to complete [5]. The agricultural sector has already seen the impact of this field's innovative solutions to problems, including precision farming, greenhouse management, and animal monitoring [6]. The IoT enabled by mobile Internet can offer practical answers to the world's problems through real-time analysis, mapping of soil characteristics, and aiding in making sound management choices [7]. The Internet of Things-enabled livestock management technologies removes the uncertainty around herd health. Battery-powered sensors on a collar or tag transmit data in near-real-time about an animal's position, temperature, blood pressure, and heart rate to the farmers' devices [8].

The use of deep learning techniques and various machine learning techniques has provided superior results when compared with conventional methods for image processing and classification [9]. Farmers can check on their crops from anywhere thanks to deep learning algorithms that monitor soil temperature and moisture levels Agriculture is crucial to the survival of all human endeavors. The world's ability to provide enough food to its inhabitants is threatened by significant challenges like overpopulation and resource competition [10]. Innovations in smart farming and precision agriculture provide helpful resources for tackling the complex difficulties in agricultural production systems that threaten agricultural sustainability [11]. The potential of UAVs for use in precision agriculture is enormous. But the price and the simplicity of controlling UAVs for smart farming could play a significant role in encouraging farmers to use UAVs. Most UAVs are operated via radio signals sent from afar [12,13]. Fertilizers and insecticides are used correctly to improve plant quality. Unmanned aerial vehicles (UAVs) are extravagant inventions that may be used to apply fertilizer and insecticides [14]. Using remote sensing technologies, UAV-IFM can detect and contain disease outbreaks by identifying and monitoring environmental changes that may indicate an out-break is occurring. Furthermore, authorities can use UAV-IFM to map and track the spread of an outbreak, allowing for rapid and precise identification of affected areas and populations.

UAV-IFM can also monitor the flow of goods and people, thereby reducing the economic and epidemiological toll. In addition, UAV-IFM can give authorities immediate feedback on the health of affected populations and the success of containment efforts, allowing them to fine-tune their response accordingly. Last but not least, UAV-IFM can be used to collect and analyze data on the success of public health interventions, providing crucial insight to authorities as they seek to comprehend the nature of the disease, its rate of spread, and the best means of preventing future pandemics.

Agriculture advocates an Integrated Farm Management (IFM) strategy that uses smart agricultural technology to increase productivity while decreasing animal production's en-vironmental effects, bolstering rural areas' economic health [15,16]. Integrated considera-tion of these systems and related externalities, the consequences of climate change may be understated, or unexpected results from adaptation implementation may arise, such as a worsening adaptability status for particular populations or ecosystems. This paper explains and defends the need for a holistic analysis method when considering the interplay between ecosystems and agricultural adaptation [17]. Based on the above discussion, the challenge of improving smart livestock farming using Unmanned Aerial Vehicles enabled Integrated Farm Management (UAV-IFM) has been designed. Farmers can collect data and monitor crops faster using drones. This may help spot difficulties early, enabling quicker and more effective responses. Agri-drones optimize fertilizer, water, seeds, and the use of pesticides. Farmers use aerial photography to measure acreage, segment crops, and map soil. Farmers claim crop insurance using drone data. The contribution has been listed here.

The main contributions of the paper are as follows:

•Raising animals can help significantly to the sustainable management of rangelands through effective livestock husbandry.
• The development of an Internet of Things assessment index system is important to increase the efficiency of livestock farming since factors including climate change, biodiversity loss, and the inability to constantly monitor cattle can indicate indications of further serious issues.
• Smart animal farming relies on deep learning based on development that bypasses traditional channels of knowledge and innovation.
• The Unmanned Aerial Vehicles enabled Integrated Farm Management (UAV-IFM) has been designed and developed for smart livestock farming.
• The experimental result has been validated with an IoT and deep learning model regarding performance, accuracy, prediction ratio, evaluation, and efficiency.
• The remaining studies are arranged as follows: Section 2 includes a literature review of studies that evaluate the existing method, Section 3 recommends a plan for UAV-IFM and its implications, Section 4 provides experimental analysis, and Section 5 provides a conclusion and prospects.

## 2 Impacts and implications of smart livestock farming

Kumar, P et al. [18] detailed many distinct models for resilient and sustainable Climate Smart Livestock Farming (CSLF) that have been implemented in various regions of the globe. Improved husbandry techniques, such as genetic enhancement of cattle, scientific housing, optimal feeding, better herd management, and digital technology, have also been suggested as climate-wise approaches to livestock production. Extension and consulting services are crucial in promoting sustainable and climate-smart livestock farming and fighting climate change in developing countries like India, where most livestock farmers are small or marginal.

Zhang M et al. [19] introduced cattle farming as moving toward precision, sustainability, and intelligence. Precision Livestock Farming (PLF) is an essential strategy for ensuring the long-term viability of smart farms, but it is only getting off the ground and holds great promise. This research proposes a novel method of applying PLF by examining its history and technological characteristics. One of the most successful answers to PLF in smart farms is now being marketed to the livestock farming sector, and it is only a matter of time before it becomes widespread. The study discusses how W-IoT might help farm animals and some difficulties and future possibilities.

Sreedevi T. R et al. [20] proposed the potential for Digital Twins (DT) to improve sustainable agriculture is enormous. However, there hasn't been as much research or development in this area as in the manufacturing, healthcare, autonomous vehicle, or aviation industries. Sustainable and self-sufficient agriculture is crucial in light of the prevalence of natural disasters like floods and diseases. It is addressed how DTs may aid in the planning, implementation, monitoring, optimization, and upkeep of hydroponic systems. Additionally, this study provides a literature analysis on the use of DT in smart farming.

Navarro E et al. [21] detailed that producers face difficulties such as a shrinking rural labor pool and rising production expenses. Smart farming is a management strategy for farms that might help alleviate the world's food shortage using IoT. This article systematically reviews the literature on smart farming with IoT using the Preferred Reporting Items for Systematic reviews (PRISMA) approach. This analysis aims to identify the key components of smart farming with IoT, including devices, platforms, network protocols, data processing technologies, and agricultural applications. However, technological advances have enabled it to utilize data for agricultural issue prevention and improved crop diagnostics in more modern techniques.

Roukh, A et al. [22] explained the use of Cutting-Edge (CE) technology like Internet of Things (IoT) sensors, drones, and farm monitoring; data-driven farming is revolutionizing the agricultural industry. Massive amounts of valuable agri-data are generated by IoT devices and are gathered and processed in real-time with the help of cutting-edge application solutions. Smart farming uses interconnected digital tools to increase efficiency and profitability in agricultural enterprises while minimizing the environmental impact of production. While numerous industry and academic-based Smart farming solutions have been presented, applying them universally to other farms is impossible. The framework presents a generic architecture to deal with the difficulties of collecting, processing, storing, and visualizing massive volumes of data in batch and real-time settings.

Mujeyi, A et al. [23] introduced the agricultural sector as vital to the economic well-being of smallholder farmers. Still, it is more vulnerable to climate change since it is dependent on ever-changing rainfall patterns. Climate-Smart Agriculture (CSA) presents promising avenues for bolstering food security and monetary gains through higher agricultural output. The potential of CSA in maximizing agricultural output is shown by data on its influence on farmer well-being gleaned from impact studies conducted as part of the technology review.

Commonly employed in adoption effect studies, the endogenous switching regression model accounts for potential selection bias and unobserved heterogeneity. Paunova-Hubenova, E et al. [24] discussed that farms raising large herds of cattle are needed to meet the rising demand for dairy and meat products. This article highlights the importance of precision livestock farming in maximizing farm profits, product quality, animal well-being, and the long-term viability of the agricultural community as a whole. Farm Management Information Systems (FMIS) are essential for running a farm. In a nutshell, the FMIS decides how things will work around the farm regarding the organization, allocation of resources, and execution of tasks. It discusses many strategies and procedures for ensuring a farm's continued viability, sustainability, resilience, and financial success.

Chatterjee, P. S et al. [25] detailed the complement of the Live Care system; this research also introduced the Cow Disease Prediction (CDP) method, a multi-class classifier that humans aren't monitoring. By studying their unique behavioral patterns, the CDP algorithm can anticipate several illnesses in cows. It included a table listing some common cow illnesses, the symptoms that may be measured for each, and the sensors that can be used to record them inside this framework. Evaluated the suggested CDP method against advanced machine learning methods to determine its efficacy.

Jerhamre E et al. [26] introduced analysis and interview research investigating the potential benefits and challenges of using Artificial Intelligence (AI) in agriculture. The cultivation of crops, dairy, and livestock subsets of agriculture are examined. Previous studies on smart farming are critically examined in a literature study that serves as the project's basis. Technical and regulatory obstacles to ownership of data, potential cyberattacks, the need for a solid business case, and a lack of technical understanding inside the industry are some of the primary obstacles identified in the report. The report finds a global trend toward a transition to smart farming. Still, the transition rate will depend on how quickly the challenges above can be overcome.

S.Mishra et al. [27] recently discussed a paper for advanced contribution in IoT in livestock farming, potential benefits of artificial intelligence, machine learning, and the Internet of Things for the cattle industry. Digital twin technology holds great promise for improved efficiency and lower costs. The results of using digital twin technology to improve precision livestock farming, reduce costs, and increase the efficiency of management processes are highly encouraging.

Zhang, Y et al. [28] introduced integrated goat detection and counting method based on deep learning (GD&CM-DL) in order to comprehend the shrewdness and accuracy of goat breeding. To solve this issue, we use computer vision techniques to create a deep learning model that can automatically detect and count goats; specifically, we focus on the Chengdu Ma goat. It is important to note that we have enhanced the model's performance by employing a number of cutting-edge methods. When compared to the 84.26% mAP of the original YOLOv5, the 92.19% mAP achieved by the upgraded detector is a substantial increase. Our proposed method has a significantly higher average overlap rate of 89.69% compared to the 82.78% achieved by the original combination of YOLOv5 and DeepSORT.

From the above discussion, challenging characteristics such as climate change, biodiversity loss, and continuous monitoring of smart livestock farming are taken into consideration as the significance of using IoT and deep learning such as [19,21,22].

Further, this research discusses the Unmanned Aerial Vehicles enabled Integrated Farm Management (UAV-IFM), which helps to predict performance, accuracy, prediction ratio, and evaluation.

Challenges related to climate change, biodiversity loss, and constant monitoring of smart livestock husbandry have been highlighted above. There are gaps and deficiencies in our understanding, as shown by previous studies on similar themes. This study's proposed UAV-IFM, for instance, has limited predictive capabilities and cannot reliably predict performance, accuracy, prediction ratio, or assessment. In addition, there is a lack of a unified strategy in the current surveys to incorporate IoT and deep learning technologies to improve the performance of the UAV-IFM. We propose a system that uses Internet of Things (IoT) and deep learning to enhance the UAV-predictive IFM's powers, thereby resolving the aforementioned informational gaps and drawbacks. When combined, these technologies will allow the UAV-IFM to make reliable predictions about performance, accuracy, prediction ratio, and evaluation. In addition, the UAV-capabilities IFM's will be enhanced by our suggested system because it provides an effective and all-encompassing method for integrating IoT and deep learning technologies. So, our suggested approach will replace the void left by previous surveys and serve as a potent resource for predicting performance, accuracy, prediction ratio, and assessment in smart cattle farming.

## 3 Methodology

This study creates a UAV-IFM to meet the growing needs of an increasingly reliant IoT, deep learning model for their smart livestock farming. Key performance parameters in animal health, productivity, and environmental load are tracked automatically and constantly using instruments and sensors in deep learning. The capacity of a machine or robot to carry out activities is usually reserved for thinking individuals. This process includes the capacities of learning, thinking, and correcting oneself. IoT technology is anticipated to play a crucial role in increasing agricultural output to satisfy the growing need for animal feed. Smart livestock farming incorporates IoT and technologies to increase operational efficiency, maximize productivity, and reduce waste via real-time field data collection, data processing, and installation of control mechanisms. The smart greenhouse, variable rate technologies, precision farming, and smart management are just a few examples of the Internet of Things-based innovations revolutionizing agriculture. The Internet of Things (IoT) may make farms more intelligent and connected by resolving livestock-related difficulties and improving the quality and quantity of livestock output. Raising livestock is a major contributor to global warming. It is the leading cause of biodiversity loss, habitat destruction, and water pollution on a global scale, accounting for half of all greenhouse gas emissions. The details are UAV enables integrated form management, IoT faming, and Deep learning techniques is discussed below.

### 3.1 Unmanned aerial vehicles enabled integrated farm management and its discussion

The Internet of Things and deep learning are the next generations of information technology, revolutionizing current production and producer services. The use of cutting-edge information technology to address the flaws of conventional cattle production, research into alternative pathways for progress, and inspire development from the inside. Raising cattle effectively is crucial to fostering steady growth and realizing sustainable development. Thus, cattle farming are experiencing a profound transformation, and conventional agricultural management expertise is becoming more irrelevant and obsolete. Precision and intelligence in management will soon become standard in the cattle industry. The general framework of smart livestock farming is discussed below.

**Fig. 1:**
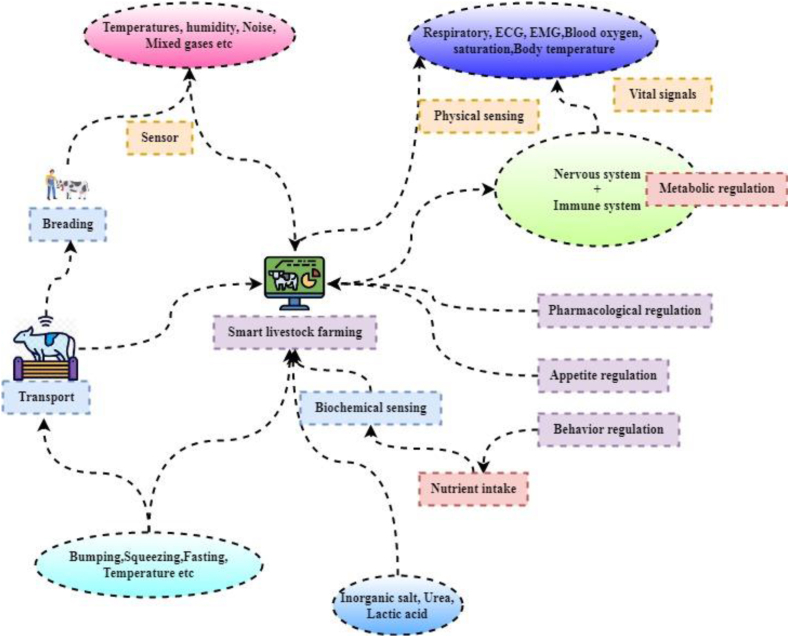
Smart livestock framing framework.

Fig. 1 illustrates the purpose of tracking vitals and reacting to environmental cues; physiological sensors must be built into various wearable devices and substrates. Now that all-organic biocompatibility technology exists, in vivo monitoring using wearable devices is a realistic possibility. However, there are many obstacles to the advancement of wearable sensors. These include, but are not limited to, selecting platforms, fabrication methods, and friendly substances that allow for easy cleaning, multiple analysis monitoring, signal mechanisms, wireless data transfer, etc. In the past, scientists have studied the many sensors in wearable devices and divided them into three broad categories: biosensing, gas sensor, mechanical sensors, micro sensors, and intelligence. The transducer in a biosensor is responsible for translating the bio-recognition event into an analytically actionable signal. “Signalization” refers to the energy transformation that takes place throughout this process.

Depending on the kind of transducer, the quantity of analyte-bioreceptor interactions will be represented by an optical or electrical signal. In order to promote positive behaviour, it is important to provide animals the freedom to explore their surroundings and the means to do so, such as enough space, environmental enrichment, and enrichment opportunities, and the freedom to make decisions about how they spend their time. Long-distance connections between immune cells and the central nervous system enable the immune system to enlist the rest of the body in the battle against infection from harmful microbes and allow the nervous system to control immune activity at the whole-organism level.

According to the “pharmaceutical regulations” are “the combination of legal, administrative, and technical measures that governments take to ensure the safety, efficacy, and quality of medicines, and the relevance and accuracy of product information. With need to encourage economic development, food security, and human livelihoods, it is important to promote the idea of a disease-free zone for certain animal illnesses. In impact, the nitrogen recycling enhances soil microbial activity and soil quality indicators including soil nutrient availability. Through the implementation of a diverse farming system and the incorporation of livestock breeds, farmers play a significant role in the preservation of biodiversity. All facets of the supply and use of livestock commodities are covered by the expression livestock systems, including the distribution and abundance of livestock, the various production strategies used for maintaining their needs, projections of current and future consumption and production, and the people involved in livestock production.

All activities and functions inside a living organism's cells need energy, and this production must be ongoing. People are feeling down, they may turn to unhealthy coping mechanisms like overeating, binge drinking, or self-harm; however, these actions rarely produce the desired results. Vital signs are indicators of the health of an individual. Healthcare providers and medical professionals routinely check for and keep an eye on these four vital signs: Internal heat, acceleration of the heartbeat, rate of respiration. In a biosensor the role of the transducer is to convert the bio-recognition event into a measurable signal. Depending on the kind of transducer, the quantity of analyte-bioreceptor interactions will be represented by an optical or electrical signal.

### 3.2 Framework IoT enabled farming

**Fig. 2:**
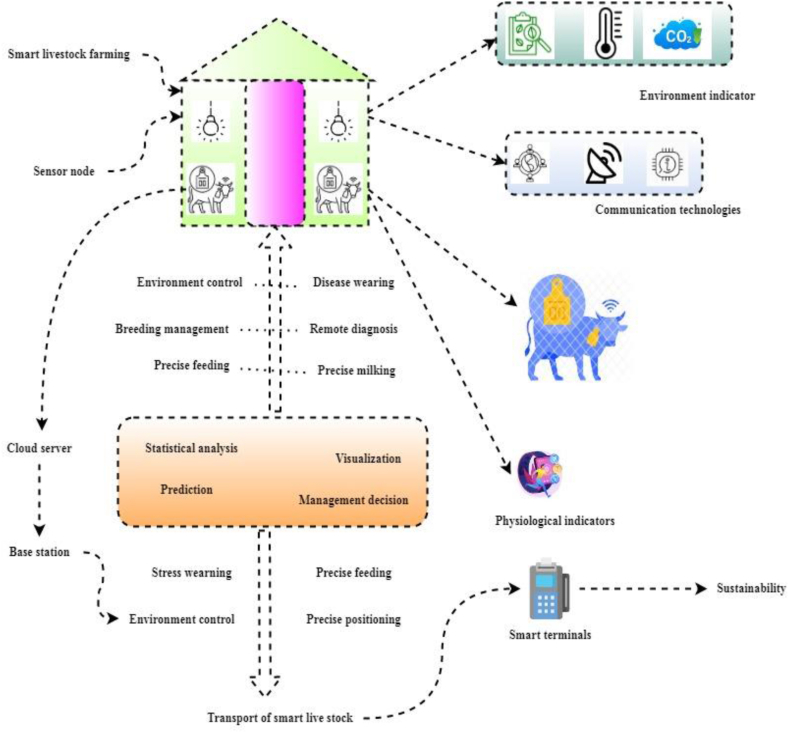
IoT in smart livestock farming.

These wearable sensors are primarily employed for vitals monitoring (e.g., Symptoms such include pulse rate, oxygen levels, warmth, and respiratory rate, etc.), behavior and movement monitoring (e.g., gait feature, etc.), and biological marker identification (e.g., glucose, lactic acid, pH, etc.) in agricultural settings (Fig. 1). Therefore, factors that mainly influence the IoT in smart livestock farming are to be predicted; then better performance is discussed as follows, Fig. 2 illustrates that wearable technology has higher standards as sensors, semiconductors, and wireless communication continues improving. Electrocardiogram (ECG) sensors, accelerometers, and other activity detection and monitoring devices are becoming common in wearables, smartphones, and other devices in the environment. Wearable technologies like gyroscopes, magnetometers, and cameras have found several applications in fields as diverse as sports and fitness tracking, health and illness diagnostics, and geolocation. There is a growing interest in developing individualized quick diagnostic equipment supported by smartphones. This tailored rapid diagnostic gadget might change the diagnosis business by combining smartphones with biosensors to make it portable, simple to wear, and inexpensive to produce. Networks of devices, federated to collect and pass data to external processing and storage platforms, provide an efficient solution to the problems described in the previous paragraph.

Fig. 2 illustrates that wearable technology has higher standards as sensors, semiconductors, and wireless communication continues improving. Electrocardiogram (ECG) sensors, accelerometers, and other activity detection and monitoring devices are becoming common in wearables, smartphones, and other devices in the environment. Wearable technologies like gyroscopes, magnetometers, and cameras have found several applications in fields as diverse as sports and fitness tracking, health and illness diagnostics, and geolocation. There is a growing interest in developing individualized quick diagnostic equipment supported by smartphones. This tailored rapid diagnostic gadget might change the diagnosis business by combining smartphones with biosensors to make it portable, simple to wear, and inexpensive to produce. Networks of devices, federated to collect and pass data to external processing and storage platforms, provide an efficient solution to the problems described in the previous paragraph.

There are several reasons that IoT holds great promise as a platform for Smart Farming. To begin, there is a massive quantity of data that must be efficiently gathered, transported, processed, and stored as a result of the need for constant monitoring and control in the agricultural sector. Secondly, as most agricultural fields provide not provide a wired energy supply and, frequently, a dependable network coverage, the deployment of extra on-field connection, enabling information exchange among IoT nodes, and the use of energy scavenging to feed them, are usually essential. Due to the global nature of livestock health problems, innovative solutions are needed, including biosensors in animal health management; wearable biosensors are generally acknowledged and recognized in animal management. The current trend in technology necessitates integrating all available sensors into a highly effective online monitoring system to track the health of animals in real-time and without lag. Appropriate health and sickness diagnostic technologies are often developed only for human use and not tested on animals. These cutting-edge tools are slowly making their way into the cattle agricultural sector.

Everything from a threshing floor to a seed vault, stables to a farmhouse. Altering environmental factors like temperature, humidity, air flow rate, etc. To better suit the needs of living organisms is another major field of study. The best way to avoid diseases acquired from contact with animals is to thoroughly wash hands with soap and water after contact with animals or their environments. Feeding livestock with precision means tailoring their nutritional intake to their specific needs as they arise. This strategy has the potential to significantly cut down on the expense and nutrient waste produced by livestock production. Urbanization and the decline of biological variety; agricultural output and the shrinking of forest areas; freshwater withdrawals and the resulting freshwater shortages; power generation and greenhouse gas emissions; all of these and more may be seen in the light of environmental indicators. In a sensor network, each node, or “sensor node,” may do some type of processing, collect sensory data, and exchange that data with other nodes in the network.

A smart terminal with a screen and keyboard, but no further commercial data processing capabilities. Smart terminals, popular in the era of minicomputers and mainframes, offered additional screen functionalities than the “dumb terminal,” such as the ability to invert the video (black on white) and show underlining. Achieving sustainability means striking a balance between economic development, environmental protection, and social well-being so that the demands of both present and future generations may be met. In a corporate setting, it refers to the measures done by upper-level executives to chart a course for future company endeavours and put concrete plans into effect. Business intelligence (BI) and analytics solutions help ensure that choices in the workplace are founded on accurate and complete information. Statistical analysis the practice of analyzing massive datasets for hidden patterns and trends. Every day, statistics are used to make judgments in academia, business, and government that are more grounded in evidence.

Despite the growing interest in wearable technology research and the breadth of its potential uses, many challenges remain. However, in PLF and smart farms using IoT; as a result, there are not enough comprehensive research and analytical mechanisms in place. Due to financial constraints, most studies and applications in animal husbandry are dispersed and rely on low-tech solutions (ear tags, pedometers, etc.) that are increasingly inadequate to fulfill well-being and social excellence standards for livestock products. Additionally, there are few professional animal testing, notably handling vast numbers of agricultural animals with precision and intelligence. Much of the present efforts in studying and implementing wearable technology have been directed toward humans. This research aims to promote the recent movement toward informatization, granularity, and smart management by examining the history and technical aspects of the IoT and then proposing an application of connected devices for reliable and long-term livestock monitoring. In Fig. 2, see a simplified version of the IoT architecture for use in smart farm livestock production for pinpoint accuracy. Therefore, from the above discussion, deep learning is introduced to solve all the problems, which is mainly smart livestock farming; it has been discussed as follows.

### 3.3 Deep learning

**Fig. 3:**
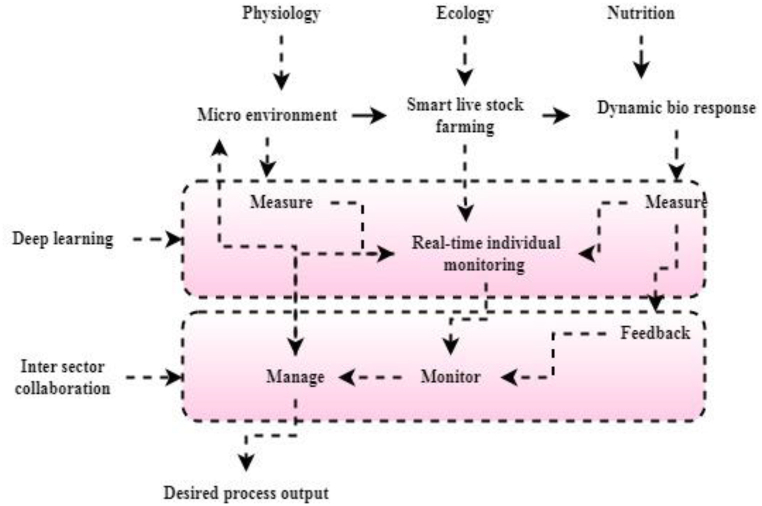
Deep learning through livestock farming.

Fig. 3 illustrates the physiology, ecology and nutrition different scenarios that impact production's economic or sustainability indices, which were an early emphasis. These streamlined techniques mirrored those seen on farms rather than attempting to communicate with the animals. As with people, technology may help animals live better lives, but the animal itself must be at the center of the process.

PLF's main goal is to provide farm-friendly technology for monitoring livestock and managing production. This involves using sophisticated data processing techniques to synthesize and mix different kinds of data, implementing up-to-date control theory to give the manufacturing process more independence, and so on. Collaboration between several scientific fields and livestock industry players is the foundation of precision livestock farming. Deep learning is used in livestock and smart agriculture to monitor crop temperature and water levels. Moreover, agricultural businesses can track their farms from wherever in the worldwide. Overall, the results show that deep learning is the basis of innovative system recognition strategies, and that the different infrastructures of various approaches provide high-quality solutions for a wide range of agricultural tasks in terms of precision and accuracy. As seen in Fig. 3, three criteria must be met inside a system to be classified as a livestock farming system.

To automatically monitor and manage animals, it is necessary to do the following: Measure animal variables (i.e., parameters relating to the animal's behavioral or physiological status) continually utilizing precise and expensive sensor systems; Know what to anticipate in terms of the changes to or the behavior of animal factors at a certain time. Every moment; Integrate predictions and online measurements in an analyzing algorithm.

### 3.4 UAV-IFM

The creation of PLF systems calls for teamwork from several fields. The collaborative PLF methodology was used to create a system that could detect the presence of a respiratory illness (the dependent variable) from a type of data sound signal by isolating the number of coughs during a particular period (the independent variable) (i.e., the process output). Fig. 3 provides a simplified schematic representation of this process. In addition to the obvious audiovisual labeling procedure, the approach uses the gold standard for explicitly tying together the two variables of interest (target and feature). The latter requires analyzing audio and video records for the presence of a set of specified “features,” such as the beginning and ending times of each sneeze.

As a result, the PLF technique requires extensive coordination across several disciplines of study, such as those dealing with animals (such as biologists, IoT, and dietitians), technicians (such as technicians, data analysts, and architects), and engineers (such as mechanical engineers). The above discussion on the construction of a smart livestock farming pathway for UAV-IFM helps to predict the IoT and deep learning as influenced by climate change, biodiversity loss, and continuous monitoring problems, as discussed: UAV-IFM activation function may be seen as the collection of transfer functions applied to produce the desired output based on input and feedback, effectively deciding the construction of smart livestock farming. The existing literature [18, 20, 24] showed that this corresponding smart livestock farming had not been effectively predicted and validated. Hence this research has included UAV-enabled IFM, which helps to predict the smart livestock farming growth factor effectively, which is discussed as follows:

Process engineering concepts are used in precision livestock farming (PLF) technology to automate agriculture in the livestock sector, enabling farmers to keep tabs on massive herds livestock farming to the employment of unmanned aerial vehicles (UAVs) for the purpose of monitoring livestock and enhancing productivity. With the help of embedded sensors, deep learning technology ensures the safe and reliable tracking of animal products all the way from farm to fork, which is a major benefit in the fight against the spread of disease and the resulting economic and public health losses.

Animal producers' output includes not just what they sell but also what they buy, what they employ in further processing, and what they consume themselves.

The IoT strategy used in smart livestock farming is based on the massive volumes of data created by farming. There are difficulties with climate change, biodiversity loss, and constant monitoring. There is need for deep learning since people in livestock farming are convinced that raising cattle intelligently would solve all of their problems. The UAV-IFM was built to validate an existing smart livestock farming platform.

**Fig. 4:**
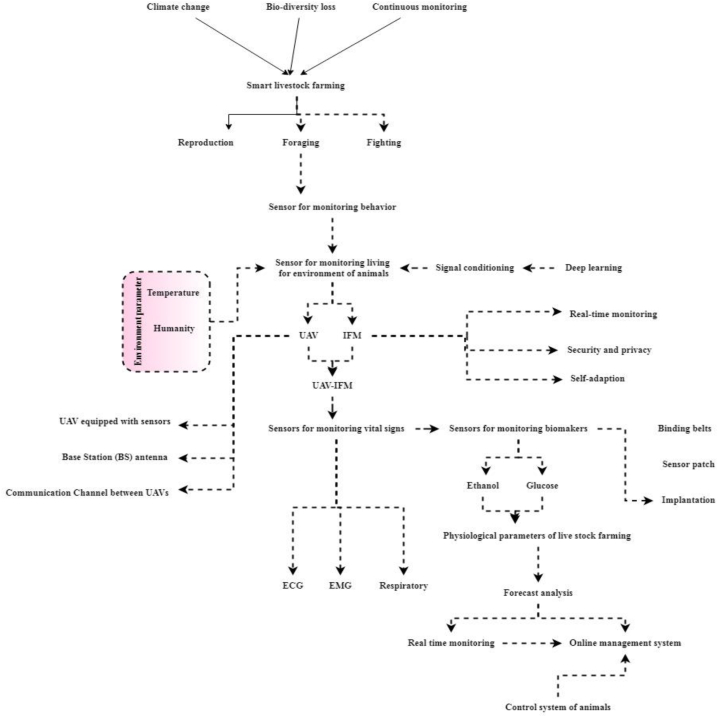
UAV-IFM.

Fig. 4 illustrates smart sensors have been integrated into wearable health management. The most crucial monitoring criteria are biochemical blood markers, but their detection and interpretation rely on expensive equipment and expert analysis. Most often, blood is drawn from a fingertip, the sample is placed on the blood sugar science test, and then readings are taken using a digital blood glucose monitor. Some electrolytic blood sugar sensors are currently on the market; these sensors are comfortable to wear and noninvasive, needing only the exterior of the epidermis to be connected to accurately quantify the venous glucose concentration. However, the methods above are rather unpleasant. Sensors that accurately monitor blood glucose levels simply by stroking skin or even entering voices may one day replace the intrusive blood-detection procedure. Non-intrusive, wearing biosensors will be useful for monitoring active livestock due to the potential difficulty handling farm animals. Wearable sensors have several real-world applications, including tracking livestock's location, activity, and biomarkers. Data fusion may be utilized to completely represent and enhance the accuracy considering the extensive mega information supplied by these detectors.

There are notable differences between inter-collision detection and conventional signal processing approaches owing to the intricate nature of the inter-providing specific clustering algorithms. Multi-source data with varying associations may be de-forged, smoothed, and fused to provide more precise and comprehensive findings; this fusion may manifest itself on several levels of detail. Finding the sweet spot between computational complexity and accuracy when arranging an inter-deployment is difficult. The main factors in maximizing productivity and sensor precision are fusion methods. Most cross-data fusion methods are optimized for specific applications to get the best possible results. An acoustic analysis strategy for cow feeding behavior was proposed, including a high pass filter, Fourier transforms, and cluster analysis to classify auditory data into feature signals of varying length, amplitude, spectrum, and energy.

Consumers used kernel principal components analysis to bolster the characteristics received after extracting them from ECG, accelerometer, and magnetometer data. UAV-IFM outcomes are utilized for surveillance and tactical planning. UAV-IFMs are categorized based on their altitude range, endurance, and weight, and can be used for a variety of purposes, including military and commercial missions. Crop cultivation and precision farming can result of unmanned aerial vehicle systems. As compared to satellite photography, drones can give real-time, high-quality aerial images over agricultural areas. Application for locating weeds and illnesses and figuring out soil characteristics and spotting differences in vegetation. The powerful characteristics were used to educate the shallow recurring model of neural networks for behavior recognition. As seen in Fig. 4, the technique by which UAV-IFM is in smart livestock farms.

Steps 1 UAV-IFM is an agri-drone that helps farmers make the most efficient use of inputs including water, fertilizer, seeds, and pesticides. Drone surveys aid farmers in determining their specific land area, dividing their crops into distinct sections, and creating detailed soil maps. In the event of agricultural damage, farmers utilize drone-collected data to file insurance claims.

Step 2 UAVs are used through geophysicists to observe underground features, geologists and geomorphologists to perform detailed DL of Earth's surface, hydrologists to observe water bodies and conduct hydrometric measurements, and meteorologists to measure weather and air quality.

Step 3 UAVs can gather data however securely transfer energy to IoT devices that are power-constrained. Most UAVs are rechargeable with higher energy storage capacity than an IoT device. The majority of IoT devices are tiny with limited battery capacity.

## 4 Result and discussion

The research concludes that the UAV-IFM effectively predicts and validates smart livestock farming compared with the IoT and deep learning method based on performance, accuracy, prediction ratio, and efficiency, which are discussed as follows.

### 4.1 Dataset description

100 images (images are collected from drones for animals Movement) are taken from 29 for this experimental analysis. Through decreased emissions and increased carbon buildup, tropical livestock farming may help in the fight against climate change.

**Fig. 5:**
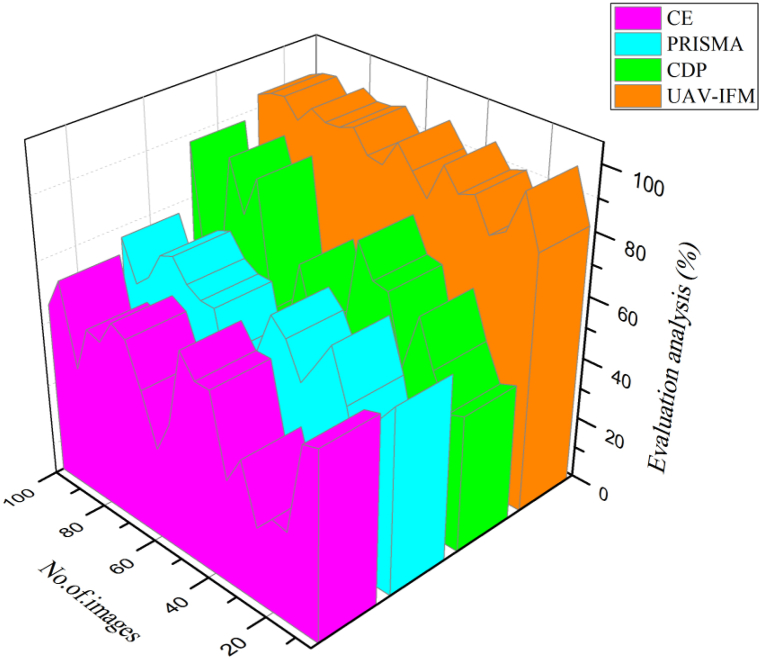
Evaluation analysis for smart livestock farming based on UAV-IFM.

Fig. 5 illustrates the evaluation analysis of smart livestock farming are crucial to ensuring a steady supply of food. In regions with a high concentration of small and medium-sized farms, it is also a major source of revenue and employment. The initiative affected government policy, which included climate-smart livestock production as a course of action for the livestock farming sector 94%. In terms of technicalities, the development of online tools to track emissions and compute climate risk and the sector's adaptability is worth noting. The engagement process with relevant stakeholders revealed the project's lack of links to the market and private sector***.*** Compared to other methods now in use, the suggested UAV-IFM is higher. According to the results of the tests, the proposed UAV-IFM is superior to the existing methods Preferred Reporting Items for Systematic reviews (PRISMA) approach [21],Cutting-Edge (CE) [22], and Cow Disease Prediction (CDP) method [25]. As a result, it's more promising than current approaches. Moreover, the proposed UAV-IFM saves both money and time, making it a more appealing option for a wide range of uses. In light of this, the proposed UAV-IFM is a workable option that provides improved performance over alternatives.

**Fig. 6:**
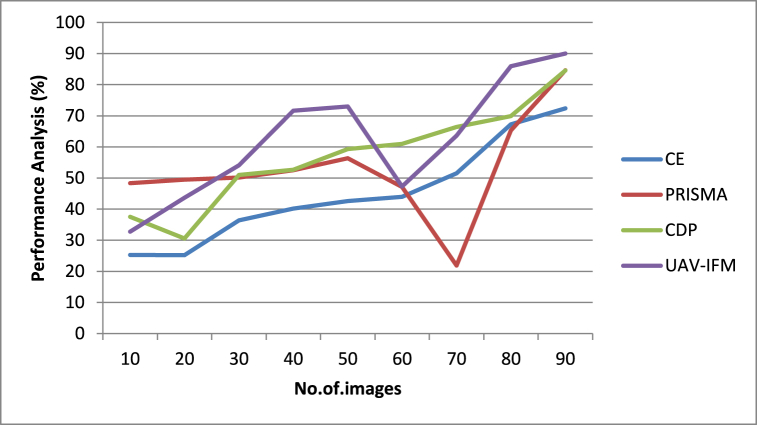
Performance analysis of smart livestock farming.

Fig. 6 illustrates that meat for animals is the input used for performance analysis of smart livestock farming-based operational models. This method leverages emerging technologies like predictive analytics and the Internet of Things, going well beyond the electronic replication of operations. The system employs self-organization and relies on pattern recognition and robust networks. The goal of “smart livestock farming,” based on the efficient use of digital technology, is to increase farm output while decreasing resource consumption and ensuring long-term sustainability. Precision livestock husbandry, in which each animal is tracked and analyzed separately. Key performance parameters in animal health, productivity, and environmental load are tracked automatically and constantly using instruments and sensors in PLF. The proposed UAV-IFM showed the highest efficiency value, a 90% improvement compared to other existing methods.

**Fig. 7:**
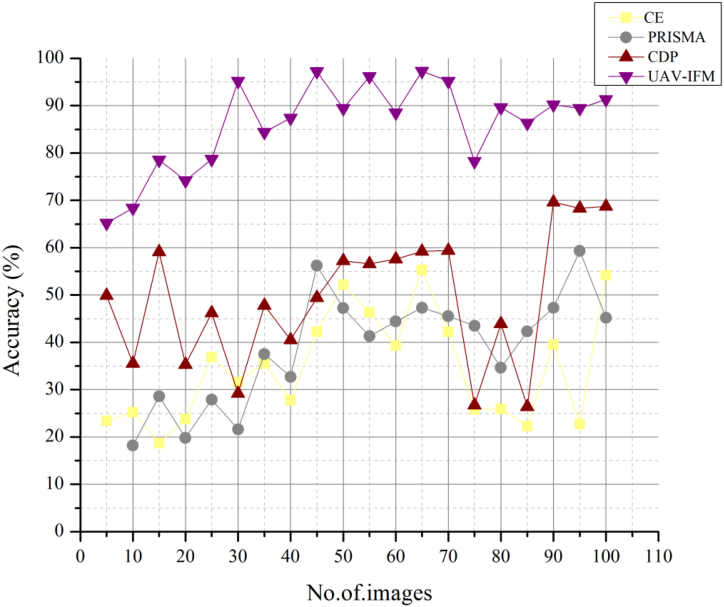
Accuracy of smart livestock farming based on UAV-IFM.

Fig. 7 illustrates food safety at home the input used for the accuracy of smart livestock farming from animals is that the former doesn't priorities metric accuracy. On the other side, “smart farming” relies on data collecting and analysis using computer technology to boost the consistency and efficiency of farming methods. Water, light, humidity, and temperature sensors for mapping soil conditions. Technologies related to communication networks and satellite positioning systems. The Internet of Things (IoT), robots, and automation all need specific hardware and software. Smart animal husbandry has the potential to reduce the environmental impact of agriculture by 91%. Leaching issues and greenhouse gas emissions may be reduced using precision agricultural systems that apply inputs such as fertilizers and insecticides where needed. Compared to other existing methods, CSLF, FMIS, and DT, the proposed method UAV-IFM is higher in accuracy ratio.

**Fig. 8:**
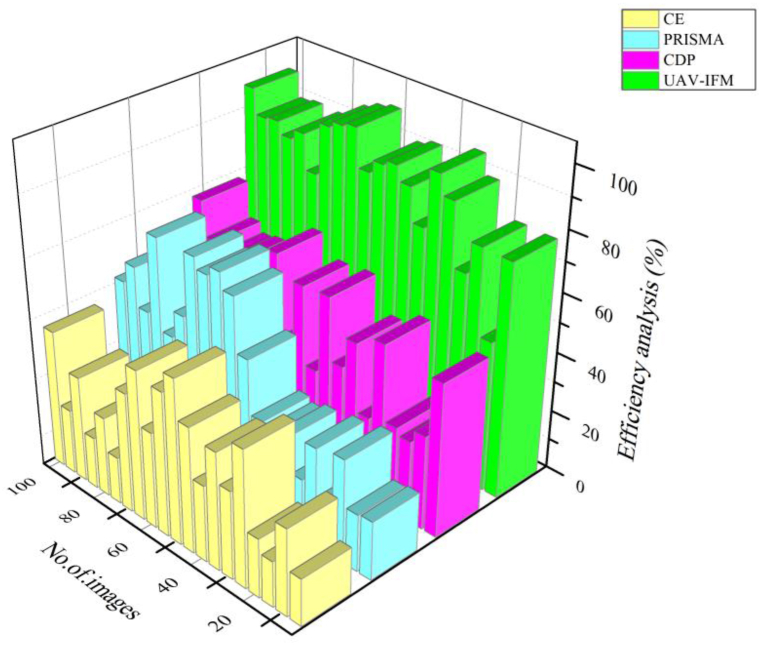
Efficiency analysis of smart livestock farming.

Fig. 8 illustrates that community nutrition is the input used for efficient analysis of smart livestock farming based on the efficient use of digital technology to increase farm output while decreasing resource consumption and ensuring long-term sustainability. Precision livestock farming, in which each animal is tracked and analyzed separately, has the most promising future). Like the management shift, modern livestock farming has adopted a new approach known as Precision Livestock Farming or Smart Farming (SF). Companies began using employee motivation and the business as a family idea to improve their operations. To effect a comparable shift in animal agriculture, PLF seeks to identify animals' requirements as early as possible and assist farmers in meeting those needs in 97%, so improving animal welfare. It is envisaged that this would improve farms overall, increasing animal agriculture's societal and economic advantages. Compared to other existing methods, CSLF, DT, and CFMIS, the proposed method UAV-IFM is higher in efficiency ratio prediction.

**Fig. 9:**
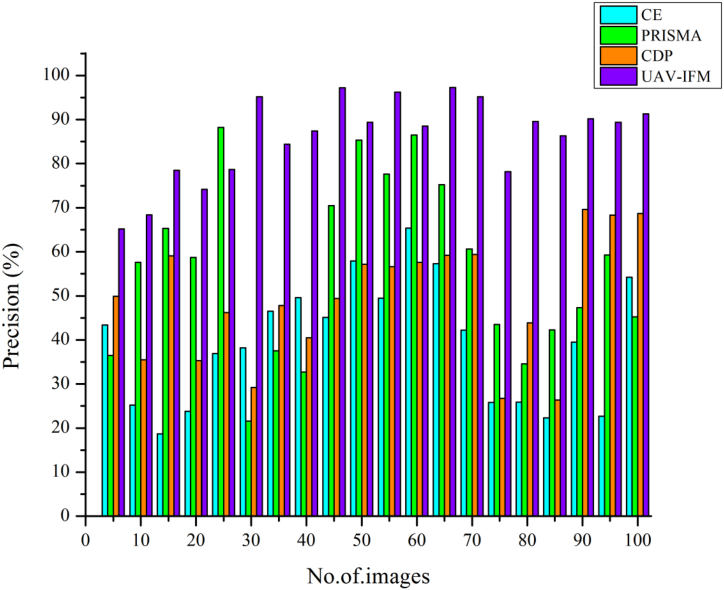
Precision of smart livestock farming based on UAV-IFM.

Fig. 9 illustrates fertilizer is the input used for predicting smart livestock farming and has the potential to improve farming significantly. Smart farming has the potential to level the playing field between industrial agriculture and subsistence farming in both emerging and industrialized nations. Water, terrain, aspect, vegetation, and soil types are just a few of the aspects that smart farming helps farmers understand better. The cattle industry across the world is quite active. It's changing because of the surging demand for meat and other animal products in emerging economies. While many production techniques are becoming more efficient and environmentally sustainable, the market for animal products in industrialized nations remains flat at 91.3%. Compared to other existing methods, CSLF, DT, and FMIS, the proposed UAV-IFM is higher in precision livestock, as shown in Fig. 9.

## 5 Conclusion

The tackles can effectively anticipate CSLF, DT, and FMIS from smart cattle farming; it is successful utilizing UAV-IFM methods, the benefits are expected accurately, and the experimental analysis is appealing. This research develops a UAV-IFM to address the rising demands of an increasingly dependent IoT, deep learning model for smart livestock farming. Key performance metrics in animal health, productivity, and environmental burden are monitored automatically and continuously using deep learning instruments and sensors. The ability of a machine or robot to do tasks is traditionally reserved for thinking beings. This process comprises the abilities to learn, think, and rectify oneself. IoT technology is expected to play a critical role in enhancing agricultural output to meet the rising need for animal feed. Smart livestock farming incorporates IoT and technologies to increase operational efficiency, maximize productivity, and reduce waste via real-time field data collection, data processing, and installation of control mechanisms. The smart greenhouse, variable rate technologies, precision farming, and smart management are just a few examples of the Internet of Things-based innovations revolutionizing agriculture. The IoT may make farms more intelligent and connected by resolving livestock-related difficulties and improving the quality and quantity of livestock output. Raising livestock is a major contributor to global warming. It is the leading cause of biodiversity loss, habitat destruction, and water pollution on a global scale, accounting for half of all greenhouse gas emissions. These issues would be greatly mitigated by switching to a plant-based diet. Industrial farming is characterized by mass output. Both cattle and grains are produced in plenty in this setting. Commercial farming needs large tracts of land, high-tech equipment, and experienced farmers to achieve desired yields. Better farm management is within reach with the help of Unmanned Aerial Vehicles and the Integrated Farm Management (UAV-IFM) system of software, sensors, and instruments. UAV-IFM is a cutting-edge method that saves time and money for farmers by keeping tabs on things like crops, livestock, and soil health. This system aids farmers in making the most efficient use of their land and in maximizing the output of their production processes. By providing data on the land and farm operations, UAV-IFM helps farmers maintain a sustainable and profitable farming operation. Unmanned technologies have been potential to transform emergency services in the future. For instance, autonomous vehicles might be utilized for transporting emergency supplies and employees and drones can be used to swiftly inspect the damage caused by natural catastrophes. An experimental evaluation of UAV-IFM shows that it can outperform smart livestock farming in terms of efficiency ratio (97%), performance (90%), accuracy (91%), and precision (91.3%).

## Author contribution statement

Shailendra Mishra: Conceptualization; Data curation; Formal analysis; Methodology; Writing – review & editing.

## Data availability statement

Data included in article/supp. material/referenced in article.

## Additional information

No additional information is available for this paper.

## Declaration of competing interest

The authors declare that they have no known competing financial interests or personal relationships that could have appeared to influence the work reported in this paper.
